# Rational design of a genome‐based insulated system in *Escherichia coli* facilitates heterologous uricase expression for hyperuricemia treatment

**DOI:** 10.1002/btm2.10449

**Published:** 2022-11-21

**Authors:** Lina He, Wei Tang, Ling Huang, Wei Zhou, Shaojia Huang, Linxuan Zou, Lisha Yuan, Dong Men, Shiyun Chen, Yangbo Hu

**Affiliations:** ^1^ CAS Key Laboratory of Special Pathogens and Biosafety Wuhan Institute of Virology, Chinese Academy of Sciences Wuhan China; ^2^ University of Chinese Academy of Sciences Beijing China; ^3^ State Key Laboratory of Virology Wuhan Institute of Virology, Chinese Academy of Sciences Wuhan China; ^4^ Hubei Jiangxia Laboratory Wuhan China

**Keywords:** gout, hyperuricemia, insulated site, microbiota, probiotic strain

## Abstract

Hyperuricemia is a prevalent disease worldwide that is characterized by elevated urate levels in the blood owing to purine metabolic disorders, which can result in gout and comorbidities. To facilitate the treatment of hyperuricemia through the uricolysis, we engineered a probiotic *Escherichia coli* Nissle 1917 (EcN) named EcN C6 by inserting an FtsP‐uricase cassette into an “insulated site” located between the *uspG* and *ahpF* genes. Expression of FtsP‐uricase in this insulated region did not influence the probiotic properties or global gene transcription of EcN but strongly increased the enzymatic activity for urate degeneration, suggesting that the genome‐based insulated system is an ideal strategy for EcN modification. Oral administration of EcN C6 successfully alleviated hyperuricemia, related symptoms and gut microbiota in a purine‐rich food‐induced hyperuricemia rat model and a *uox*‐knockout mouse model. Together, our study provides an insulated site for heterologous gene expression in EcN strain and a recombinant EcN C6 strain as a safe and effective therapeutic candidate for hyperuricemia treatment.

**Abbreviations**
CKD: chronic kidney disease
EcN: *E. coli* Nissle 1917
PCA: principal component analysis
SD: Sprague–Dawley
SPF: specific pathogen‐free
sUA: serum urate
WT: wild type

## 1 INTRODUCTION

Hyperuricemia is a highly prevalent disease characterized by elevated urate (uric acid) levels in the blood owing to purine metabolic disorders. This disease is also the most important risk factor for the development of gout.^1^, ^2^ The prevalence of hyperuricemia ranges from 10% to 20% in developed countries.^3^, ^4^, ^5^ Consequently, the incidence of gout is 5% in the United States, 4.75% in Europe, and 3.8% in Australia.^4^, ^5^, ^6^, ^7^ Importantly, many hyperuricemic patients are asymptomatic and thus clinically neglected; however, diseases caused by hyperuricemia cannot be ignored.^5^ Increasing evidence suggests that hyperuricemia is a risk factor for the development of a variety of comorbidities, including hypertension, diabetic renal disease, obesity, metabolic syndrome, fatty liver, and cardiovascular disease.^2^, ^5^, ^8^, ^9^, ^10^ Therefore, hyperuricemia remains to be a global public health issue.

Urate is a key product of the purine metabolic pathway and is highly conserved in living organisms.^11^ In most species, urate is metabolized to a more soluble compound called allantoin by urate oxidase (uricase) and is further degraded to urea or ammonia.^12^ In contrast, the uricase gene found in ancestral apes has been silenced in humans owing to evolutionary events; thus, urate is the final product of the purine metabolic pathway.^12^, ^13^ Approximately, two‐third of urate in the human body is excreted by renal urate transporters (such as GLUT9 and URAT1), while the remaining one‐third is transported by the ABCG2 transporter in the small intestine (via the extra‐renal excretion pathway) and cleared by intestinal microorganisms via a process known as uricolysis.^14^, ^15^, ^16^ The overproduction or underexcretion of urate is the main cause of hyperuricemia. Therefore, traditional pharmacological urate‐lowering therapies (ULTs) target urate generation (xanthine oxidase inhibitors)^6^, ^17^ or renal urate excretion (uricosurics)^18^ or directly increase urate degradation (uricase).^19^, ^20^ However, these drugs have potential severe adverse effects and are not recommended for a large proportion of patients with asymptomatic hyperuricemia.^21^

The intestinal tract plays an increasing role in urate excretion, particularly in patients with chronic kidney disease (CKD), whose renal elimination of urate is impaired.^19^, ^22^ The reduction of extrarenal urate excretion is a common cause of hyperuricemia in patients with CKD.^23^, ^24^ According to previous studies, dysbiosis of intestinal flora exists in patients with gout and serum urate (sUA) levels are associated with gut microbiome changes.^25^, ^26^ Therefore, modulation of the gut microbiota is an alternative approach to hyperuricemia treatment.^27^ Appropriate supplementation of probiotics plays a role in urate lowering by regulating the intestinal flora.^3^, ^28^ Moreover, urate concentration in intestinal has been found to be positively related to sUA, and oral administration of uricase can reduce sUA in hyperuricemic rats and urate oxidase‐deficient mice,^19^, ^21^, ^29^ suggesting that the use of engineered probiotics expressing functional uricase is an attractive strategy for the treatment of hyperuricemia.

The intestinal microbiota is associated with the metabolic health of the human host and possess tremendous potential in the field of biotherapeutics delivery for the treatment of various human diseases.^30^, ^31^ *Escherichia coli* Nissle 1917 (EcN) is a probiotic with superior intestinal adaptation^32^, ^33^ that has been modified as a bacterial “living factory” for various applications. As a result, several recombinant strains have been used in clinical trials.^34^, ^35^, ^36^ However, for clinical development of engineered bacterial therapeutics, the safety of orally administered chassis and genetically stable are crucial to bacterial pharmacokinetics in vivo.^35^, ^37^ Thus, bacterial genome editing is a reliable strategy and undoubtedly can reduce burden caused by plasmids, particularly in biomedical applications.^38^ However, irrational loci on the genome can not only decreases performance but also interfere with native transcription of bacterial chassis.^39^ Thus, ultra‐stable genetic editing approach based on the genome without affecting background gene expression would strengthen the clinical applications of recombinant strains.^38^

In this study, we provide a programmable approach of genome‐based highly insulated expression system to facilitate biotherapeutics delivery, and engineered a strain called EcN C6 with insulated expression of uricase from *Cyberlindnera jadinii* for the treatment of hyperuricemia. We characterized that the fusion of the uricase gene with the TAT signal peptide FtsP is essential for efficient degradation of urate by the strain. Further, we demonstrated the effects of EcN C6 in alleviating hyperuricemia and related symptom and restoring the gut microbiota disturbed by hyperuricemia in rat and mouse models. Collectively, our data suggest that EcN C6 is a safe and effective therapeutic candidate for hyperuricemia.

## 2 RESULTS

### 2.1 EcN expressing uricase in periplasmic space in effectively degrades urate in vitro

**FIGURE 1:**
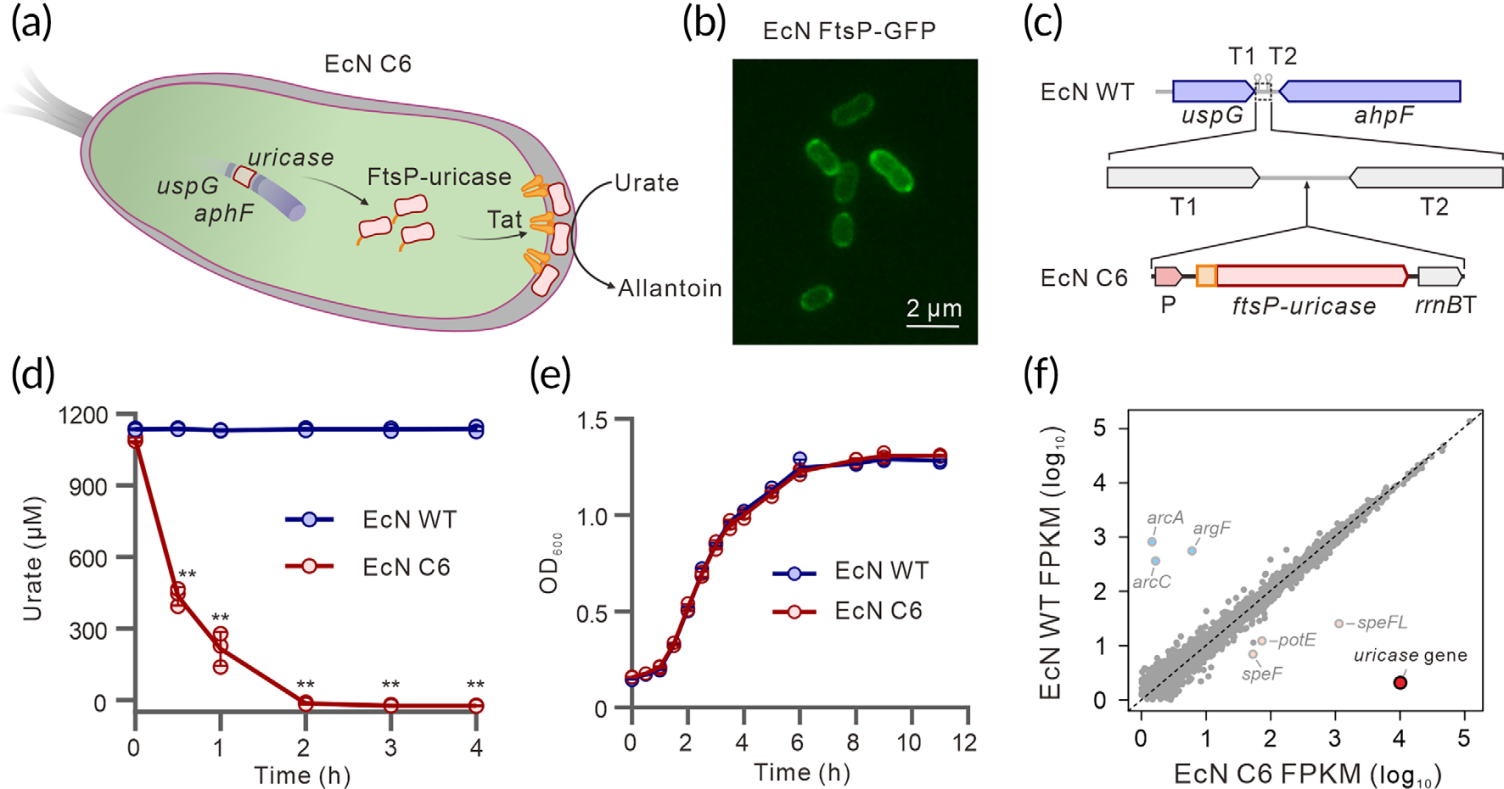
Periplasmic expression of uricase in engineered EcN C6 to degrade urate in vitro. (a) Schematic of the engineered strain, EcN C6, with periplasmic expression of uricase by degradation of urate to allantoin. (b) Periplasmic localization of GFP fused with the FtsP signal peptide. (c) Insulated site for insertion of exogenous fragment in the EcN genome. (d) Urate degradation activity of EcN C6 in vitro. (e) Growth comparison of EcN WT and EcN C6 strains. (f) Linear plots of gene expression in EcN WT and C6 strains as tested by RNA‐seq analysis. Data represent measurements from three independent bacterial cultures, bars show the mean ± SD using two‐tailed unpaired Student's *t*‐test (**p < 0.01).

We first aimed to test whether the expression of uricase in EcN degrades urate in vitro (Figure 1a). To achieve this purpose, we expressed the uricase gene from *C. jadinii*, which catalyzes the oxidation of urate to allantoin, in the cytoplasmic space of EcN using a P15A originated plasmid. In vitro urate degradation assay revealed that the degradation efficiency is extremely low (Figure S1a). To explore whether the expression location of uricase would affect the efficiency in urate degradation, we next fused the uricase gene with different secretion signal peptides, but most of these signal peptides could not enhance uricase activity (Figure S1a). Interestingly, when the uricase gene was fused with a TAT secretion signal peptide FtsP (SufI), the urate degradation efficiency was greatly improved (Figure S1b), and fluorescence localization of GFP showed that the FtsP signal peptide drives GFP protein into bacterial periplasmic space compared with the control group (Figure 1b and S1c).^40^ Consistent with the location of the periplasmic space, the supernatant from FtsP‐uricase strain did not show uricase activity, suggesting that there was no uricase leakage into the supernatant (Figure S1d). Together, these results indicate that fusion of the FtsP signal peptide with uricase is vital for engineering EcN strain to degrade urate.

### 2.2 Construction of insulated expression system for heterologous uricase delivery

Next, to construct an engineered strain without affecting global gene expressions, we aimed to select a suitable site for exogenous gene integration in the EcN genome. By comparing RNA‐sequencing reads of EcN under different conditions,^41^, ^42^, ^43^ we identified a noncoding region between two 3′‐end face‐to‐face located genes *uspG* and *ahpF*, whose RNA reads are extremely low, but the surrounding coding regions are highly transcribed (Figure S2a). Prediction of RNA secondary structure of 3′‐end of both *uspG* and *ahpF* genes showed two opposite ρ‐independent terminator structures named T1 and T2, respectively (Figure S2b), suggesting that the region between these two terminators would be an ideal “insulated site” candidate and widely found in the *Enterobacteriaceae* (Figure S2c). At this site, we inserted a uricase‐expressing cassette containing a synthesized σ^70^‐dependent promoter, a coding region carrying the FtsP signal peptide in fusion with the uricase gene, and a *rrnB* terminator (*rrnB*T), into this insulated site to obtain an engineered strain named EcN C6 (Figure 1a–c). Compared with EcN wild‐type (WT), EcN C6 degraded urate in vitro within 2 h (Figure 1d), but showed similar growth pattern as demonstrated by its growth curve in normal condition (Figure 1e) and survival in MU medium (Figure S3a). Furthermore, competitive growth assay showed the ability of EcN C6 to kill *Salmonella Typhimurium* LT2 under low iron conditions was comparable as EcN WT (Figure S3b), suggesting that the probiotic characteristics of EcN C6 were not affected by functional uricase expression. Transcriptomic analysis showed only six genes were significantly affected by uricase expression, while the global transcriptional profiling of EcN C6 was not influenced (Figure 1f). Together, we successfully engineered a recombinant EcN strain with insulated expression of periplasmic uricase to degrade urate in vitro.

### 2.3 EcN C6 ameliorates hyperuricemia disease in rat model

**FIGURE 2:**
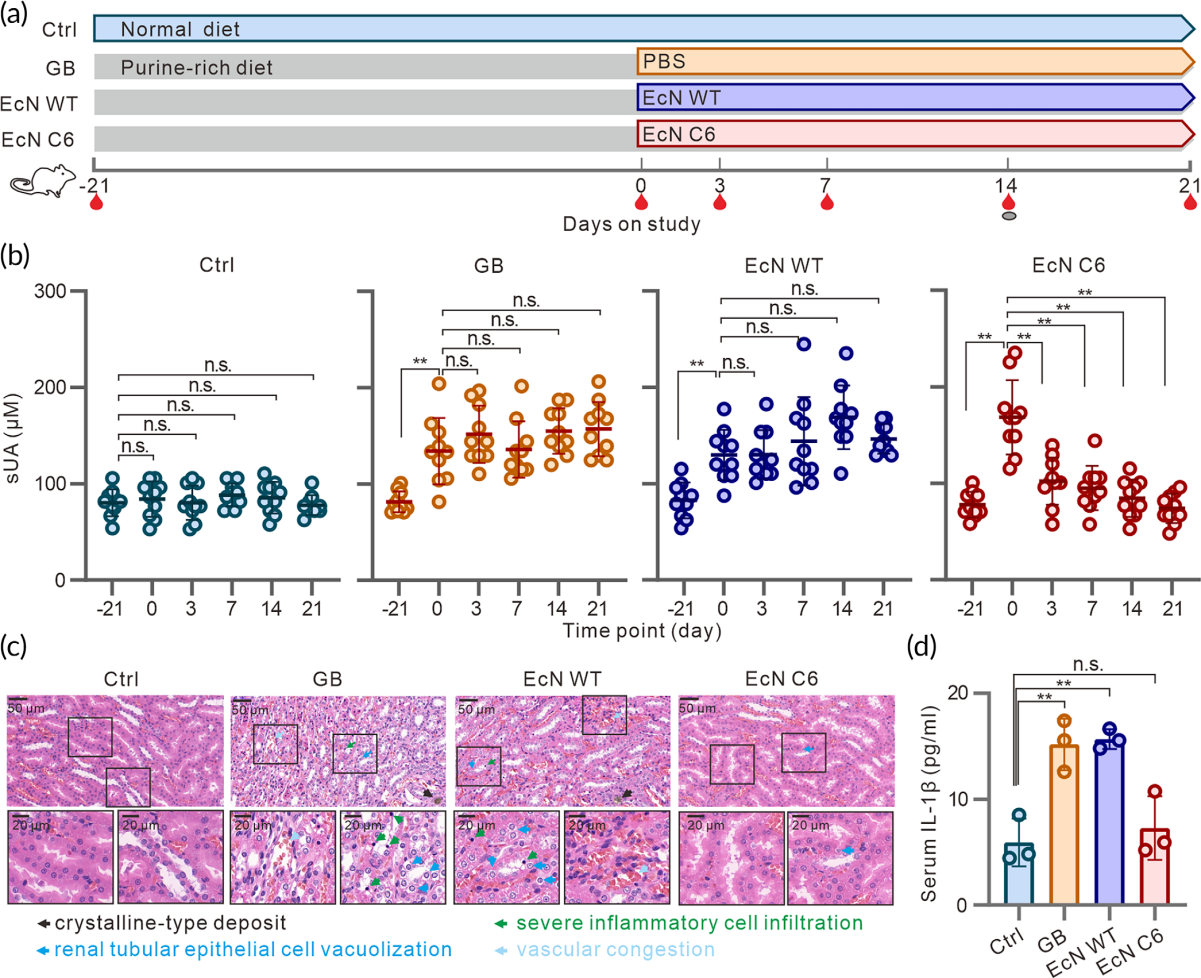
Treatment with EcN C6 decreases sUA levels and alleviates kidney damage in a rat model. (a) Schematic representation of the treatment of recombinant EcN C6 expressing uricase in a rat model of hyperuricemia. Male SD rats (*n* = 40) were divided into four groups (10 for each group); three quarters were pretreated with purine‐rich food for 21 days to induce hyperuricemia and the remaining one quarter was used as the control with no treatment (Ctrl). The EcN WT, EcN C6 (3 × 10^10^ CFU for each strain), or gavage buffer (GB) was administered orally to the hyperuricemic rats for another 21 days. Purine‐rich food was also provided during this treatment to maintain high sUA level. At indicated time points, bloods and feces were collected. Rats were euthanized after 21 days of treatment for kidney imaging. (b) sUA levels of purine‐rich food hyperuricemia rats after treatment with different strains. (c) Representative renal tissue sections with hematoxylin and eosin staining. Scale bars, 50 μm or 20 μm. (d) IL‐1β levels in rats with different treatments. Statistical analysis was performed using two‐tailed unpaired Student's *t*‐test (**p < 0.01).

To investigate the efficacy of EcN C6 in vivo, we applied a purine‐rich food‐induced hyperuricemia rat model to determine whether EcN C6 can degrade urate in animal model. Briefly, we first treated specific pathogen‐free (SPF) Sprague–Dawley (SD) rats with purine‐rich food for 21 days to induce hyperuricemia. Subsequently, the rats were orally administered EcN WT or C6 with purine‐rich food (Figure 2a). In this model, the sUA levels in rats increased significantly after 21 days of purine‐rich food induction (Figure 2b). Importantly, daily treatment with EcN C6 successfully decreased sUA levels within 3 days after the first administration, whereas the EcN WT or gavage buffer (GB) did not decrease sUA levels in this hyperuricemic rat model (Figure 2b).

To further explore whether EcN C6 could alleviate the pathological symptoms caused by hyperuricemia,^44^, ^45^ rats were euthanized 21 days after treatment. Compared with the chow diet group, the EcN C6 group displayed attenuated urate‐induced inflammation, as demonstrated by detailed renal pathologies of HE staining and serum IL‐1β levels (Figure 2c,d). Groups administered GB or the EcN WT strain showed significant renal crystals, severe inflammatory cell infiltration, and large amount of vacuolation in renal tubular epithelial cells and interstitial congestion of renal tubules, even significantly increased serum IL‐1β, while the EcN C6 group displayed attenuated urate‐induced inflammations of kidney, compared with the chow diet group (Figure 2c,d). Besides, the urate‐associated inflammation reflected by IL‐6, TNF‐α and diamine oxidase (DAO) levels in serum of hyperuricemia rats were also alleviated (Figure S4). Together, these data illustrate that EcN C6 possesses urate‐lowering effects and alleviates hyperuricemia symptoms in a rat model, suggesting that the EcN C6 strain is applicable for the treatment of hyperuricemia.

### 2.4 EcN C6 alleviates dysbiosis of the gut microbiota in hyperuricemia rats

**FIGURE 3:**
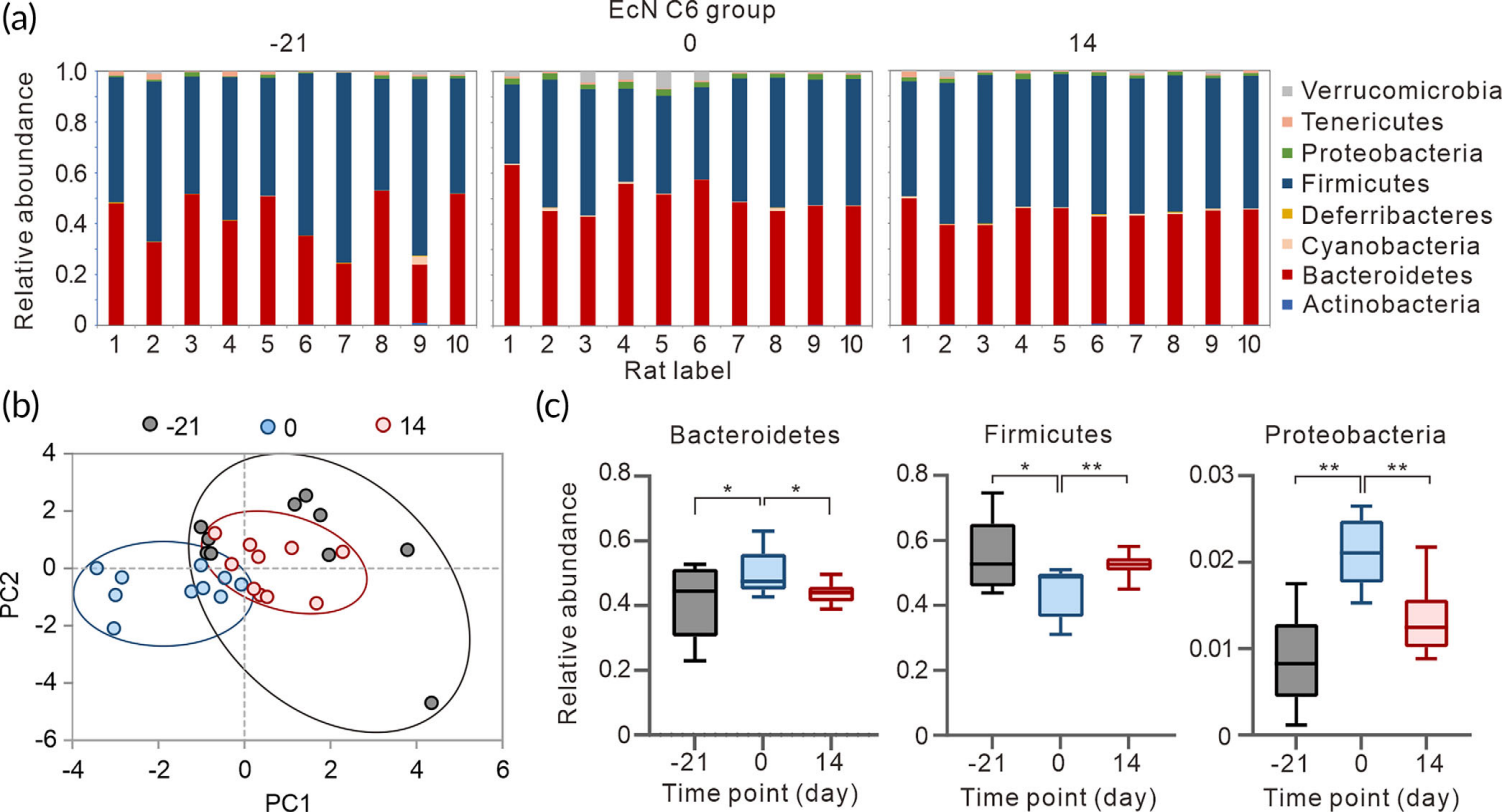
EcN C6 alleviates gut microbiota dysbiosis in hyperuricemia rats. (a) Comparison of phylum relative abundance of rats before (−21) and after (0) high‐purine food induction, as well as treatment with the EcN C6 strain for 14 days.^14^ (b) Principal coordinates plot of the gut microbiota in rats treated with EcN C6 at different time points as in A. (c) Relative abundance of *Bacteroidetes*, *Firmicutes*, and *proteobacteria* in the gut microbiota of the EcN C6 group at −21, 0, and 14 days (*n* = 10). Statistical analysis was performed using two‐tailed unpaired Student's *t*‐test (**p < 0.01).

To explore the effect of EcN C6 on gut microbes in a hyperuricemia rat model, we extracted fecal bacterial DNA from rats before and after 21 days of purine‐rich food induction, as well as 14 days after EcN C6 treatment in the purine‐rich food‐induced hyperuricemia model. 16S rRNA gene amplicon sequencing was then employed to detect bacterial species in rats at different stages, which revealed that *Bacteroidetes* and *Firmicutes* were the two most abundant gut microbial phyla (Figure 3a). Principal component analysis (PCA) demonstrated that the gut microbial composition changed significantly in the hyperuricemia rat model (Day 0 compared with Day −21) (Figure 3b). Further, statistical analysis revealed that hyper urate induced gut flora disorders, including alterations in the contents of *Bacteroidetes*, *Firmicutes*, and *Proteobacteria* (Figure 3c–e), as well as *Verrucomicrobia* and *Deferribacteres* (Figure S5), whereas treatment with EcN C6 alleviated dysbiosis in the gut microbiota (Figure 3). Overall, these results suggest that EcN C6 may balance the gut microbiota in rats with hyperuricemia.

### 2.5 Administration of EcN C6 twice per week lowers sUA levels

**FIGURE 4:**
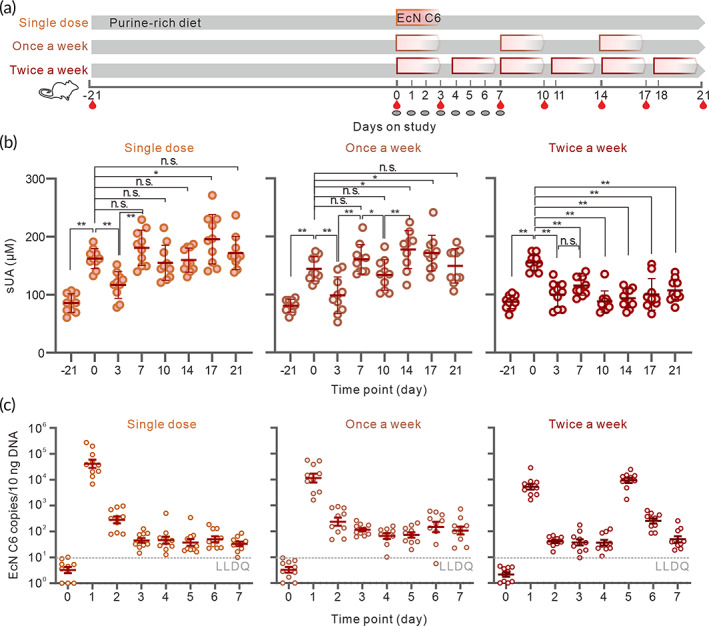
Administration of EcN C6 twice per week is required to lower sUA levels. (a) Schematic representation of the treatment of hyperuricemia rats with different doses of EcN C6. Male SD rats (*n* = 30) were pretreated with purine‐rich food to induce hyperuricemia and divided into three groups: Single‐dose group (1.2 × 10^11^ CFU), once per week group (3 × 10^10^ CFU) and twice per week group (3 × 10^10^ CFU). Blood samples and feces were collected at indicated time points. (b) sUA levels of hyperuricemia rats administered purine‐rich food after treatment with different doses of the EcN C6 strain. (c) Detection of EcN C6 copies in feces at different time points (*n* = 10 for each group). Statistical analysis was performed using two‐tailed unpaired Student's *t*‐test (**p < 0.01).

To explore the effective dosage of EcN C6 for lowering the sUA level, we treated the hyperuricemic rats with different doses of EcN C6 (Figure 4a). As expected, sUA levels in hyperuricemic rats were significantly decreased 3 days after a single‐dose administration of EcN C6 but were elevated after 7 days (Figure 4b). Similarly, once a week dose treatment resulted in the same trend (Figure 4b). The limited effect of once per week dose of EcN C6 in lowering sUA levels may be caused by limited resident time of the strain in the gut, as EcN C6 could be detected only within 2 days in feces after each administration (Figure 4c), which is consistent with previously reports.^34^, ^46^ On the contrary, the group dosed with EcN C6 twice a week obviously increased the residence of EcN C6 in the gut and successfully lowered the sUA levels during the treatment period (Figure 4b,c), which further supports our conclusion that the colonization of EcN C6 in the gut is important for lowering urate levels. The discrepancies between different dosage group were also reflected by the renal pathology HE staining, creatinine, urea nitrogen, and cytokines levels in serum of hyperuricemia rats (Figure S6).

### 2.6 EcN C6 alleviates hyperuricemia symptoms in a *uox*‐knockout mouse model

**FIGURE 5:**
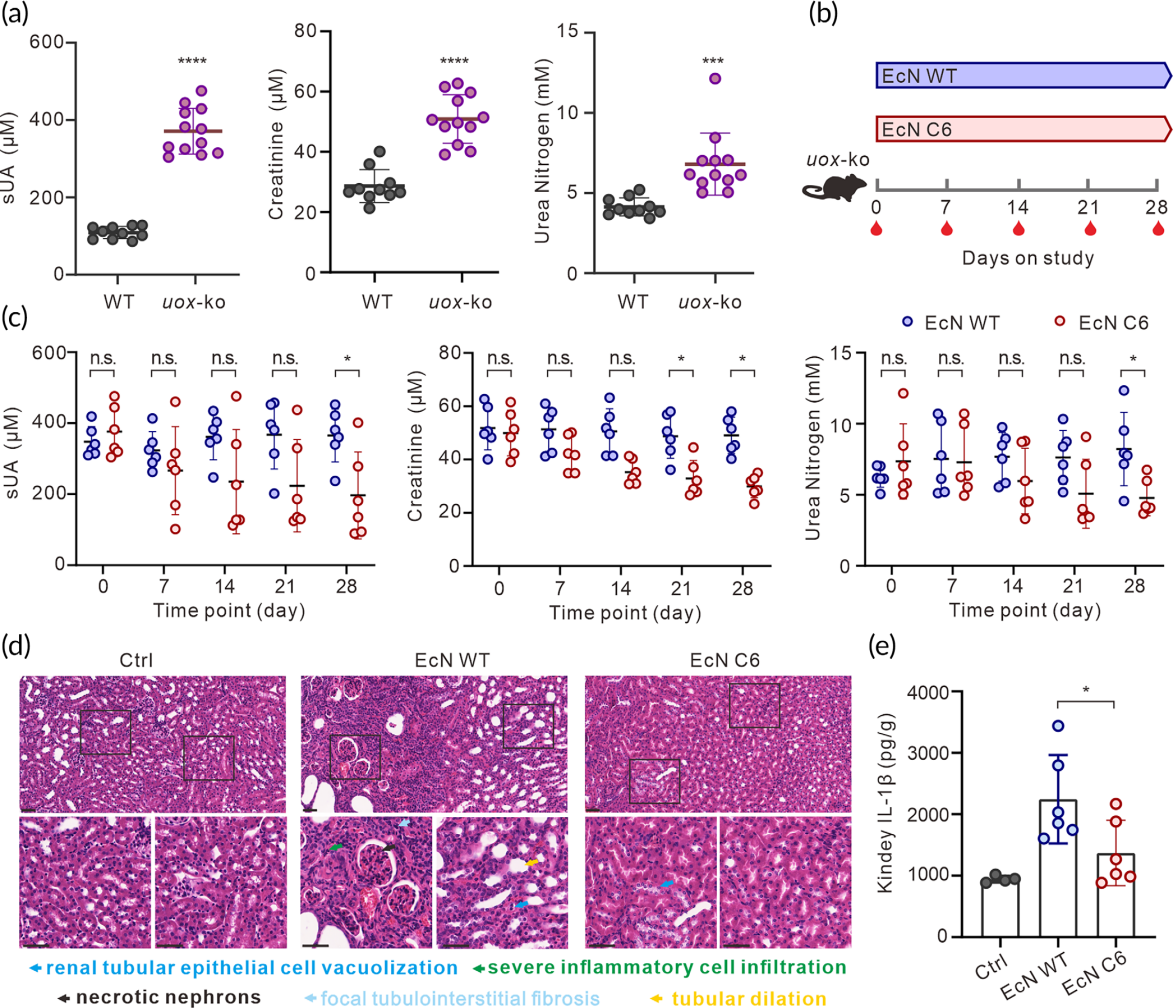
Effects of EcN C6 treatment on hyperuricemia symptoms in a *uox*‐ko mouse model. (a) Serum urate, creatinine, and urea nitrogen levels of wild‐type (*n* = 10) and *uox*‐ko (*n* = 12) mice. (b) Schematic representation of the treatment with EcN WT (1 × 10^10^ CFU) or EcN C6 (1 × 10^10^ CFU) for 1 month in the *uox*‐ko mouse model. Blood samples were collected at indicated time points. After treatment for 28 days, the kidneys were dissected for tissue HE staining and inflammatory factor detection. (c) Serum urate, creatinine, and urea nitrogen levels in mice administered EcN WT or EcN C6 at indicated time points (six for each group). (d) Representative renal tissue sections with HE staining. Scale bars, 50 μm. (e) Kidney IL‐1β levels in wide‐type mice (Ctrl, *n* = 4) or *uox*‐ko mice administered EcN WT or EcN C6 treatment (six for each group). Statistical analysis was performed using two‐tailed unpaired Student's *t*‐test (*p < 0.1; ***p < 0.001).

We employed a *uox*‐knockout mouse model to further confirm the effect of EcN C6 on lowering sUA levels. As knockout of the *uox* gene is detrimental,^47^ only 12 knockout mice were obtained. Stably elevated sUA, serum creatinine, and urea nitrogen levels in these mice indicated that the model had been successfully established (Figure 5a). Mice were then divided into two groups and treated with EcN WT and EcN C6 (Figure 5b). In contrast to the WT group, the EcN C6 group had significant alleviation of the hyperuricemia indicators, including sUA, creatinine and urea nitrogen (Figure 5c). Similar to our observations in the rat model, EcN C6 treatment lowered the inflammatory response as reflected by the renal pathology HE section (Figure 5d) and kidney IL‐1β levels (Figure 5e). Taken together, our data suggest that EcN C6 can alleviate the symptoms of hyperuricemia in a *uox*‐knockout mouse model.

## 3 DISCUSSION

**FIGURE 6:**
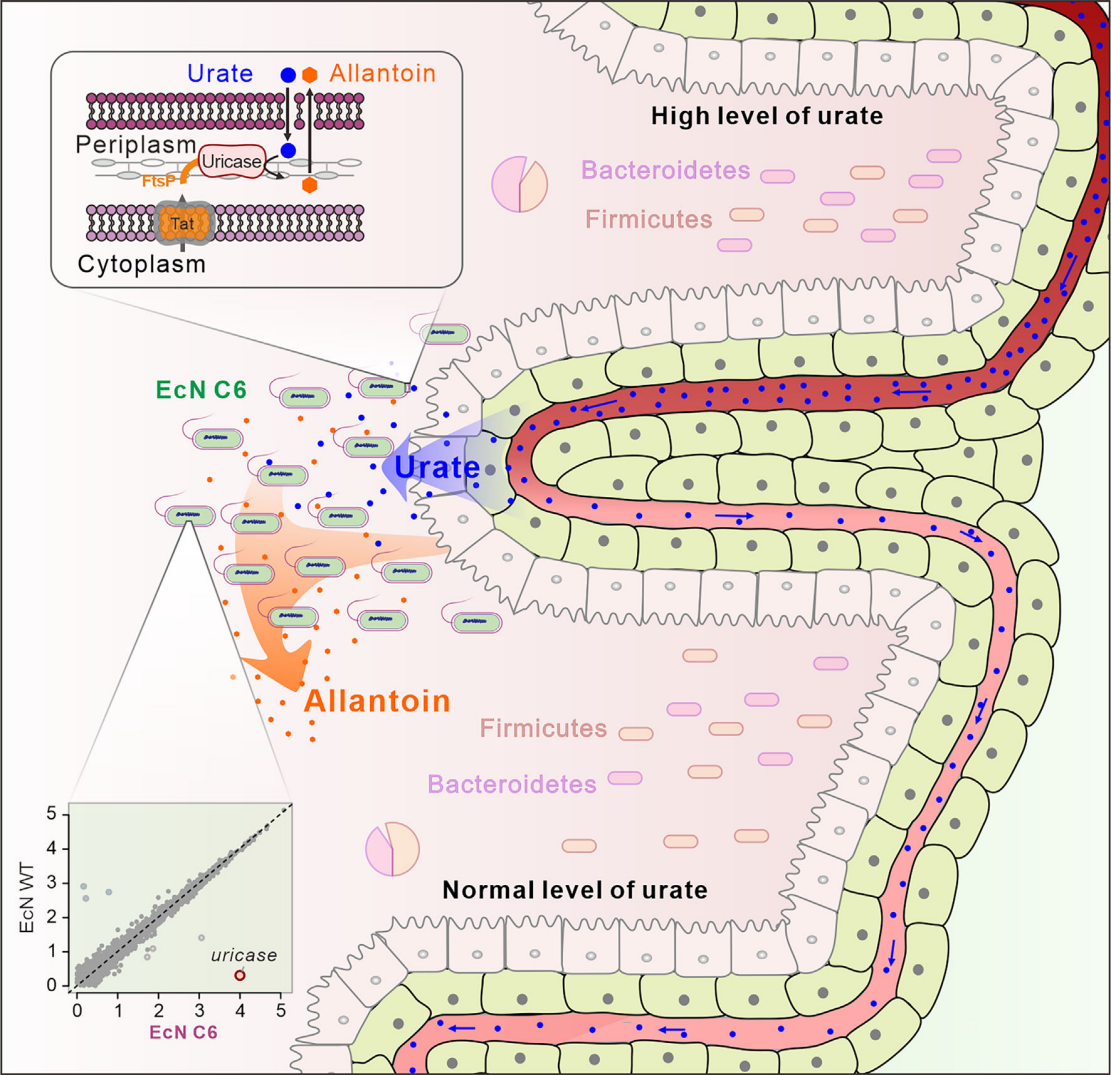
Proposed model for the action of EcN C6 strain in lowering sUA levels in vivo. In a hyperuricemia model, urate in the blood (blue dots) is transferred to the intestine by urate transporters. Engineered EcN C6 (green ellipses) with insulated expression of periplasmic uricase shows similar global transcription profile as the parent strain (down insert), and successfully degrades urate into allantoin (up insert) in the gut to lower sUA levels. Meanwhile, the administration of EcN C6 alleviates dysbiosis of the gut microbiota in the hyperuricemia model.

Gout is a common and challenging health issue worldwide. Despite the availability of treatments for lowering urate levels, these drugs mainly aim to inhibit urate synthesis or promote urate excretion, thereby placing a remarkable burden on the kidneys.^23^, ^48^ Previous studies showed that elevated level of urate causes kidney damage by promoting autophagy, and induces β‐cell injury via the NF‐κB‐iNOS‐NO signaling axis,^49^, ^50^ and may have side effects on gut bacteria.^51^, ^52^, ^53^ Here, as summarized in Figure 6, we successfully engineered a probiotic strain, EcN C6, with insulated expression of periplasmic uricase to directly degrade urate and alleviate the symptoms and dysbiosis of the gut microbiota caused by hyperuricemia, ultimately providing an efficient and friendly method for the rapid treatment of hyperuricemia.

In addition to traditional urate‐lowering chemical drugs, enzymes related to the degradation of urate are gaining attention, and may serve as a more direct method for hyperuricemia treatment.^54^, ^55^ Clinical data have shown that modified uricases, such as pegolase, lablipase, and pregabalin, display excellent performance in the treatment of intractable gout disease by intravenous injection; however, their duration of action in vivo is limited. Notably, large amounts of supplementation can induce antibody production and are cost‐effective.^56^, ^57^ Uricases are strongly not recommended as first‐line therapy by the American College of Rheumatology guidelines for the management of gout owing to their limited duration of action.^58^ To overcome the limitation, we engineered a probiotic EcN C6 expressing periplasmic uricase (Figure 6). Probiotic EcN is the preferred microbial synthetic biology vector and can successfully colonize in the small intestine,^46^ which plays an essential role in regulating urate levels.^29^ Moreover, colonization of EcN near the epithelial cells in the small intestine allows them to easily access to urate that is transported from the blood^29^ and obtain oxygen^46^, ^59^ as a necessary substrate for uricase function. This strategy has markedly extended the application of uricase for hyperuricemia treatment.

The location of the uricase expressed in *E. coli* is essential for its ability to degrade urate. Although *aegA* and *ygfT* can degrade urate under microaerobic or anaerobic conditions in *E*. *coli*,^60^ in contrast to uricase, this effect was almost negligible both in vitro and in vivo (Figures 1d and 2b). In addition, there are 10 nucleobase‐ascorbate transporter (NAT) family‐related proteins in *E. coli* that are responsible for transporting different forms of base metabolites. Further, the *ygfU* gene located at the inner membrane was hypothesized to import urate^61^ and overexpression of YgfU could improve the urate degradation efficiency in *E. coli*.^62^ However, our data showed that cytoplasmic expression of uricase in the EcN strain only slightly decreased the urate level in vitro (Figure S1b), suggesting that the efficiency of urate import may be limited by the bacterial cell membrane or the affinity of urate transporters. Consistently, a previous study showed that *E. coli* expressing cytoplasmic uricase did not show significant enhancement in urate degradation.^63^ While, *E. coli* expressing secreted uricase could be functional in hyperuricemia rat model, although its effect in lowering sUA is limited.^63^

The EcN C6 expressing periplasmic uricase driven by FstP signal peptide significantly enhanced its activity in urate degradation. In our study, we found that fusion of uricase with the Tat secretory signal peptide FtsP, which transports folded proteins across biological membranes,^64^ successfully enabled the EcN strain to degrade urate in vitro and in vivo (Figures S1b and 2b), while fusing with the Sec secretory signal peptides (OmpA and TamA), which translocate unfolded proteins across the cytoplasmic membrane,^64^ did not show such effect (Figure S1b). These data suggest that the uricase folding events in cytoplasmic is important for its activity.

Our EcN C6 design included a feature to insulate uricase expression. As a safe and common bacterial vector, EcN has been adopted for the regulation of mucosal immunity,^65^, ^66^ metabolic diseases^34^, ^35^, ^67^ and pathogenic infections.^68^, ^69^, ^70^ However, the expression patterns for these functional proteins are mainly based on recombinant plasmids, which fail to comply with the FDA's requirements for live biotherapeutic products.^71^ Previous studies have attempted to insert exogenous genes near the *malEK*, *malPT*, and *yicS/nepI* genes in EcN^34^, ^72^; however, these types of genomic editing may interfere with native gene transcription.^34^, ^38^ As the design of ultra‐stable genetic editing in *E. coli* is important for living therapeutics,^38^ herein, we have demonstrated that a method for searching insulated sites on the bacterial genome by analyzing previous transcriptome data of EcN under different conditions^34^, ^41^, ^42^ and proposed an insulated expression system for the exogenous gene. Concretely, we identified a natural dual‐terminator structure between *uspG* and *ahpF* on the *E. coli* genome, providing an ideal insulated expression site, which is widespread in *Enterobacteriaceae* (Figure S2c). Importantly, our RNA‐seq data revealed that this “insulated site” ensures insulated expression of the inserted uricase as expression of only six genes were significantly changed comparing between the EcN C6 and parent strains. Therefore, our study presents an insulated site, which is effective and valuable for the recombinant expression of other target genes in EcN or similar bacterial chassis in the field of synthetic biology.

Influenced expression of six genes associated with arginine and ornithine metabolism pathways in EcN C6 strain may not influence its application in hyperuricemia treatment. In EcN C6 strain, the inserted uricase could catalyze urate to allantoin, which is further degraded to ammonia. The ammonia could be further utilized as the nitrogen source to synthesize ornithine or arginine.^73^ Consequently, the *acrA‐acrC‐argF* operon, which is associated with conversion of arginine to citrulline and ammonia,^74^ would be repressed due to the accumulation of ammonia. In contrast, the *speFL‐polE* operon, which is responsible for ornithine capture to control polyamine synthesis,^75^ would be activated due to the accumulation of ornithine. Nevertheless, the expressions of other genes related to the arginine or ornithine metabolism pathway, the bacterial growth and the probiotic phenotype were not influenced in EcN C6 strain (Figures 1f,e and S3). Therefore, we conclude that the EcN C6 could be a candidate for the treatment of hyperuricemia.

## 4 CONCLUSION

In this study, we first analyzed the published transcriptome data to identify an “insulated” site located between the *uspG* and *ahpF* genes in the EcN genome; we next applied homologous recombination to insert a cassette expressing periplasmic uricase at this insulated site to obtain an engineered strain named EcN C6. In vitro urate degradation assay and global RNA‐seq were subsequently applied to confirm the activity and insulated expression of uricase in EcN C6, respectively. Importantly, the recombinant probiotic EcN C6 strain showed strong ability in decreasing serum urate levels and relieving symptoms in hyperuricemia murine models, thus offering great potential in clinical application. Together, our study provides an insulated site for heterologous gene expression in EcN strain and an engineered EcN C6 strain as a safe and effective therapeutic candidate for hyperuricemia treatment.

## 5 MATERIALS AND METHODS

### 5.1 Bacterial strains

The bacterial strains used in this study are listed in Table S1. *Escherichi*a *coli* and *S. Typhimurium* LT2 strains were grown in Luria‐Bertani (LB) medium or agar (LBA) at 37 °C and supplemented with 50 μg/ml kanamycin, 100 μg/ml ampicillin, or 30 μg/ml chloramphenicol, when necessary. EcN WT and C6 were prepared in LB and the cell pellets were re‐suspended in protective buffer (15% v/v glycerol, 5% w/v trehalose, and 10 mM MOPS, pH 7.3) and frozen at −80°C until use, as previously described.^34^

### 5.2 Plasmid constructions

The plasmids and oligonucleotides used in this study are also listed in Table S1. To express uricase fused with different signal peptides, a uricase gene from *C. jadinii* (GenBank: XM_020212619.1) with a signal peptide coding region from either *ftsP* (N‐terminal 30 aa, GenBank: NP_417489.1), *ompA* (N‐terminal 27 aa, GenBank: NP_415477.1), *tamA* (N‐terminal 27 aa, GenBank: NP_418641.1), *lpp*‐*ompA* (N‐terminal 29aa of *lpp* and 46aa‐159aa of *ompA*, GenBank: NP_310411.1 and NP_415477.1), *yebF* (N‐terminal 118 aa, GenBank: NP_416361.2), or *inpNC* (N‐terminal 211aa and C‐terminal 99aa, GenBank: AF013159) was cloned into the pKT100 plasmid^76^ using a ClonExpress II One Step Cloning Kit (Vazyme, China). The *fstP*‐*gfp* expression clone was constructed in a similar manner by replacing the uricase gene with *gfp* fragment.

### 5.3 Fluorescence imaging

To verify the periplasmic localization of the FtsP‐GFP fusion protein, the EcN strain transformed with pKT‐*ftsP*‐*gfp* was grown at 37°C in LB liquid medium containing Kan (50 μg/ml) to OD_600_ ~ 0.3. Cells were collected by centrifugation at 5000*g* for 3 min and resuspended in phosphate buffered saline (PBS). The samples were dropped onto a slide and fixed by slight heating. Fluorescence images were obtained using a fluorescence microscope (OLYMPUS).

### 5.4 EcN C6 strain construction

To construct the EcN C6 strain, a fragment containing the synthesized promoter P6, a signal peptide encoded by *ftsP*, the uricase gene from *C. jadinii*, and the *rrnB*T terminator was inserted between *uspG* and *ahpF* homologous fragment, and cloned into the suicide pDM4 plasmid, which carries chloramphenicol resistance gene and sucrose sensitive *sacB* gene.^77^ The resulting plasmid was transformed into *E. coli* S17‐1 cells. Transconjugation^77^ was performed using this *E. coli* S17‐1 and EcN carrying the temperature‐sensitive pKD46 plasmid^78^ as the donor and recipient cell, respectively. The single cross clones were selected using LB with Amp (100 μg/ml) and Chloramphenicol (30 μg/ml) plate, and the double cross clones were selected in LB plate containing 15% sucrose. The pKD46 plasmid in EcN C6 was removed by culturing the strain at 42°C. The insertion of the uricase fragment into the EcN C6 strain was confirmed by PCR and DNA sequencing.

### 5.5 Urate degradation assay by EcN strains

Overnight cultures of the EcN strains were diluted 1:100 with 3 ml fresh LB liquid medium, incubated at 37°C until OD_600_ ~ 0.6 (0.3 mM IPTG was added when necessary), and continually incubated until OD_600_ ~ 1.0. Cell pellets were collected by centrifugation and resuspended in an equal volume of MU medium as previously described.^15^ Urate concentration in the medium was monitored using A_293_ absorption with NanoDrop One (Thermo Fisher Scientific, USA) at the indicated time points.^15^ The standard curve between the A_293_ values and urate concentrations was examined to quantify the urate concentrations in the MU medium.

### 5.6 Growth assay of EcN C6

Freshly streaked EcN WT and EcN C6 colonies were inoculated with 5 ml of LB and grown overnight at 37°C. Cultures were transferred at a ratio of 1:100 into fresh 5 ml of LB and grown for 12 h at 37°C. The OD_600_ was measured at the indicated time (BioTek). Equal amounts of EcN WT and EcN C6 were incubated in MU medium for 1 h, and the CFU of strains was counted by 10‐fold serial dilution in LB plate. Competitive growth for EcN and *S. Typhimurium* LT2 under iron‐rich or iron‐limiting conditions was performed as previously described.^70^

### 5.7 RNA‐seq analyses

To characterize the transcriptional profiles of EcN WT and EcN C6 strains, triplicate cultures of each strain were incubated overnight in LB broth at 37°C. Overnight cultures were then diluted 100‐fold in 4 ml LB broth and incubated for 2.5 h. RNA was extracted using TRIzol reagent, as described in the manufacturer's protocol (Invitrogen, USA). rRNA in the extracted RNA was removed using a Ribo‐off rRNA Depletion Kit (Vazyme, China). The RNA library was constructed using the NEBNext® Ultra II RNA Library Prep Kit for Illumina (NEB, USA) and sequenced using the Illumina HiSeq X Ten platform.

### 5.8 Hyperuricemia rat model

Two‐week‐old SPF SD rats were purchased from Beijing Vital River Laboratory Animal Technology Co., Ltd. After acclimatization for 1 week, rats were divided into groups (10 per group) and were either fed with high‐purine food containing maintenance powder, 10% yeast (OXIFOD) and 0.1% adenine (Sangon, China) to induce hyperuricemia,^79^ or normal chow as a control.

EcN WT and EcN C6 were grown overnight in LB at 37°C with shaking. Overnight cultures were used to inoculate 1 L of LB in 3 L baffled flasks, cultures were grown with shaking at 37°C for 5 h. Cell pellets were obtained by centrifuge at 4500 *g* for 30 min and resuspended in protective buffer (15% v/v glycerol, 5% w/v trehalose, and 10 mM MOPS, pH 7.3). The strains were adjusted to 3 × 10^10^ CFU/ml or 1.2 × 10^11^ CFU/ml and frozen at −80°C until use.

After induction by high‐purine food for 3 weeks, rats were administered 1 ml of gavage buffer (GB group), EcN wild‐type (EcN group, 3 × 10^10^ CFU per dose), or EcN C6 (EcN C6 group, 3 × 10^10^ CFU per dose) for 3 weeks. To explore the minimum dose of EcN C6, similar approaches were employed, except that 1.2 × 10^^11^ CFU was used for the single‐dose group. Serum samples were collected at the indicated time points to test sUA, creatinine, and urea nitrogen levels using commercial kits, according to the manufacturer's instructions (Jiancheng, China). After treatment with these strains for 21 days, two rats in each group were euthanized by slow asphyxiation with CO_2_. The left kidney was dissected for tissue HE staining.

### 5.9 *Uox*‐knockout mouse model

Conventional SPF C57BL/6J^*uox/uox*^ (*uox*‐ko) mice purchased from the Shanghai Model Organisms Center Inc. (SMOC) were maintained and bred at the Center for Animal Experiments at the Wuhan Institute of Virology. Allopurinol (90 μg/ml) was added to enhance the survival of newborns when administered to the mother and withdraw 1 week before the experiment. Mice were divided into two groups of equal sex and age. In this model, the EcN WT or C6 strain was administered daily by oral gavage 0.2 ml strains (6 × 10^9^ CFU per dose). Serum was collected at the indicated time points after treatment for 28 days. Mice were euthanized by slow asphyxiation with CO_2_. The left kidney was dissected for HE staining, and the right kidney was used for tissue homogenization to determine the inflammatory factors.

### 5.10 ELISA

Serum was isolated from the blood of rats and mice by low‐speed centrifugation (1000 g, 10 min). Suspensions from ground kidney samples were collected by low‐speed centrifugation (2000 g, 15 min). To detect the cytokines in serum or kidney, the samples were analyzed using a rat or mouse IL‐1β ELISA Kit (Neobioscience, China), rat IL‐6 ELISA Kit (Neobioscience, China), rat TNF‐α ELISA Kit (CUSABIO, China), rat DAO ELISA Kit (CUSABIO, China), following the manufacturer's instructions.

### 5.11 16s rRNA library preparation and sequencing

Feces collected from hyperuricemic rats were frozen at −80°C until use. DNA was extracted using the E.Z.N.A. Stool DNA Kit (OMEGA, USA) following the manufacturer's instructions. The 16S rRNA gene (V4 region) was amplified by two‐step PCR enrichment using barcodes for multiplexing.^80^ Pooled DNA was purified using AMpure XP beads (Beckman, USA). DNA libraries were constructed using the NEBNext Ultra II FS DNA Library Prep kit (NEB, USA) and sequenced using the Illumina HiSeq X Ten platform.

### 5.12 Quantification of EcN C6 colonization

To quantify the colonization of EcN C6, qPCR was performed to determine the copy numbers of the EcN *fimA* gene in 10 ng of fecal genomic DNA using iTaq Universal SYBR Green Supermix (Bio‐Rad, USA). Standard curves were constructed by quantitatively testing 10,^8^ 10,^7^ 10,^6^ 10,^5^ 10,^4^ 10,^3^ 10,^2^ 10,^1^ and 10^0^ copies of EcN C6 genomic DNA according to a previously described protocol.^3^ All measurements were performed in triplicate.

### 5.13 Statistical analysis

Statistical significance between two groups was analyzed by unpaired Student's *t*‐test (two‐tailed) using GraphPad Prism 8 or the R package (version 3.2.2).

## AUTHOR CONTRIBUTIONS

**Lina He:** Conceptualization (equal); investigation (equal); methodology (equal); visualization (equal); writing – original draft (equal); writing – review and editing (equal). **Wei Tang:** Investigation (equal); methodology (equal). **Ling Huang:** Investigation (supporting). **Wei Zhou:** Visualization (supporting). **Shaojia Huang:** Investigation (supporting). **Linxuan Zou:** Investigation (supporting). **Lisha Yuan:** Investigation (supporting). **Dong Men:** Conceptualization (supporting); methodology (equal). **Shiyun Chen:** Conceptualization (equal); supervision (equal); writing – review and editing (equal). **Yangbo Hu:** Conceptualization (lead); funding acquisition (lead); methodology (equal); project administration (lead); supervision (equal); visualization (equal); writing – original draft (equal); writing – review and editing (lead).

## CONFLICT OF INTEREST

The authors declare that they have no conflict of interest. The WIV has filed patents on EcN C6 strain construction and application, which are based in part on the work reported here.

### PEER REVIEW

The peer review history for this article is available at https://publons.com/publon/10.1002/btm2.10449.

## Supporting information


**Appendix S1:** Supporting information

## Data Availability

RNA‐sequencing and 16s rRNA gene sequencing reads were submitted to the NCBI Sequence Read Archive (SRA) under accession: PRJNA818111 and PRJNA818085, respectively. The data that support the findings of this study are available from the corresponding author, Y.H., upon reasonable request.
