# Supplementary material for: Rational design of a genome‐based insulated system in Escherichia coli facilitates heterologous uricase expression for hyperuricemia treatment
Source: Bioeng Transl Med. 2022 Nov 21;8(2):e10449. doi: 10.1002/btm2.10449 (PMC10013758; doi:10.1002/btm2.10449)
Supplement: Supplementary file 1 — Appendix S1: Supporting information [file BTM2-8-e10449-s001.docx]

**Supporting information**

**Rational design of a genome-based insulated system in *E. coli* facilitates heterologous uricase expression for hyperuricemia treatment**

Lina He^1,3,#^, Wei Tang^1,^^#^, Ling Huang^1,3^, Wei Zhou^1,3^, Shaojia Huang^1,3^, Linxuan Zou^1,3^, Lisha Yuan^1,3^, Dong Men^2,*^, Shiyun Chen^1,*^, Yangbo Hu^2,*^

^1^ *CAS Key Laboratory of Special Pathogens and Biosafety, Wuhan Institute of Virology, Chinese Academy of Sciences, Wuhan, 430071, China*

*^2^ State Key Laboratory of Virology, Wuhan Institute of Virology, Chinese Academy of Sciences, Wuhan, 430071, China*

^3^ *University of Chinese Academy of Sciences, Beijing,* *100049, China*

^#^ These authors contributed equally to this work

^*^ For correspondence:

D.M. d.men@wh.iov.cn, S.C. sychen@wh.iov.cn or Y.H. ybhu@wh.iov.cn

**Supplementary Table**

**Table S1** Strains, plasmids and primers used in this study.

| Name | Description | Source or application |
| --- | --- | --- |
| Strains |  |  |
| EcN | *E. coli* Nissle 1917 (serotype O6:K5:H1) | Lab collection |
| EcN C6 | Recombinant EcN strain with insulated expression of uricase between *uspG* and *ahpF* genes. | This study |
| S17-1 | *thi pro hsdR*^–^ *hsd*M^+^ *recA* RP4 2-Tc::Mu-Km::Tn*7* | Lab collection |
| DH5α | Strain used for clone construction | Lab collection |
| *Salmonella* *Typhimurium* LT2 | *Salmonella enterica* serovar *Typhimurium* LT2 (wild-type *S*. *Typhimurium* LT2) | Lab collection |
| Plasmids |  |  |
| pKT100 | Cloning vector, p15A replicon, Kan^R^ | ^77^ |
| pKT-*ompA*-*uricase* | Plasmid expressing OmpA-uricase, Kan^R^ | This study |
| pKT-*tamA*-*uricase* | Plasmid expressing TamA-uricase, Kan^R^ | This study |
| pKT-*ftsP*-*uricase* | Plasmid expressing FtsP-uricase, Kan^R^ | This study |
| pKT-*lpp*-*ompA*-*uricase* | Plasmid expressing Lpp-OmpA-uricase, Kan^R^ | This study |
| pKT-*yebF*-*uricase* | Plasmid expressing YebF-uricase, Kan^R^ | This study |
| pKT-*inpNC*-*uricase* | Plasmid expressing InpNC-uricase, Kan^R^ | This study |
| pKT-*ftsP*-*gfp* | Plasmid expressing FtsP-GFP, Kan^R^ | This study |
| pDM4 | Suicide vector for recombinant strain construction, Cm^r^ | ^78^ |
| pDM4-*UAm*-C6 | Vector of insertion UAm-C6 fragment | This study |
| Oligonucleotides |  |  |
| *ftsP*-Forward | CAATTTCACACAAGAAGGAGATCACATATGTCACTCAGTCGGCGTCAG | EcN C6 inserted fragment |
| *ftsP*-Reverse | GTACTTCGTTGTCATTGGATTGCCCGGCTGCGCTGGCCTTC |  |
| *uricase*-Forward | TCCAATGACAACGAAGTACCTGGTTCCATGACTGCCACCGCAGAAACCTC |  |
| *uricase*- Reverse | GCAGTCGATCGTACGCTACTAGCAGAATCCGGCGATGTTC |  |
| *P6*- Forward | AGTATATACACTCCGCTAATGTGAGTTAGCTCACTCATTAGGCACCCCAGGCTTGACA |  |
| *P6*- Reverse | CTCCTTCTTGTGTGAAATTGCACACATGCTAGGAGCCGATGATTAATTGTCAAGCCT |  |
| *rrnBT*- Forward | CGAACCTGAACCACTACCATAAAACGAAAGGCCCAGTCTTTCGAC |  |
| *rrnBT*- Reverse | TAGCGTACGATCGACTGCCAGGCATCAAATAAAACGAAAGGCTCAGTCGAAAGACTGG |  |
| *uspG*- Forward | CTAGCGGAGTGTATATCAAGACCAGCCAGAAGCTGCTGGCGA | pDM4-UAm-C6 construction |
| *uspG*- Reverse | TAGCGGAGTGTATATACTGGCATTAAAAAGCCCTGCAGGGATGGCTCCGG |  |
| *ahpF*- Forward | GGTAGTGGTTCAGGTTCGCAATAAAAAAGCCGCCAGGTTTGAC |  |
| *ahpF*- Reverse | CAGGTTACCCGCATGCAAGACGACGTTGATGTGATCGACAGC |  |
| *gfp*- Forward | TCCAATGACAACGAAGTACCTGGTTCCAGCAAGGGCGAGGAGCTGTTCA | pKT-*ftsP*-*gfp* construction |
| *gfp*- Reverse | CGAACCTGAACCACTACCTTACTTGTACAGCTCGTCCATGCC |  |
| *ompA*-Forward | CAATTTCACACAAGAAGGAGATCACATATGAAAAAGACAGCTATCGCGAT | pKT-*ompA*-*uricase* construction |
| *ompA*-Reverse | GTACTTCGTTGTCATTGGAGGTGTTATCTTTCGGAGCGGCCTG |  |
| *tamA*-Forward | CAATTTCACACAAGAAGGAGATCACATATGCGCTATATCCAACAGTTAT | pKT-*tamA*-*uricase* construction |
| *tamA*-Reverse | GTACTTCGTTGTCATTGGACTGTAGACGGACGTTCGCGG |  |
| *lpp*-*ompA*-Forward | CAATTTCACACAAGAAGGAGATCACATATGAAAGCTACTAAACTGGTACTGG | pKT-*lpp*-*ompA*-*uricase* construction |
| *lpp*-*ompA*-Reverse | CAAAGCCAACATACGGGTTAATTCCCTGATCGATTTTAGCGTTGCTGGA |  |
| *yebF*-Forward | CAATTTCACACAAGAAGGAGATCACATATGAAAAAAAGAGGGGCGTTTTTAGGGC | pKT-*yebF*-*uricase* construction |
| *yebF*-Reverse | GTACTTCGTTGTCATTGGAACGCCGCTGATATTCCGCCAT |  |
| *inpNC*-Forward | CAATTTCACACAAGAAGGAGATCACATATGACTCTCGACAAGGCGTTGGTG | pKT-*inpNC*-*uricase* construction |
| *inpNC*-Reverse | CCACCACTGCCGCCACCTTTAACCTCGATCCAATCATCATCTTCG |  |
| *fimA*-Forward | ATACTACGACGGTAAATGGT | qPCR for examining the copy numbers of EcN C6 genome |
| *fimA*-Reverse | CGGCTTTTGTGGCAACAGTGG |  |
| *uox*-Forward 1 | CAACTCAACAGGAAAGAGATCCAG | Identification of C57BL/6J *uox*-knockout mouse |
| *uox*-Reverse 1 | GTGTTGCCGCCATCTCTGCCTCTAGGC |  |
| *uox*-Forward 2 | GTAATAACAGGATAGAGTCTCCTCGG |  |
| *uox*-Reverse 2 | GGATGAATGCATGGACGTGTTTGATCCC |  |

**Supplementary Figures**


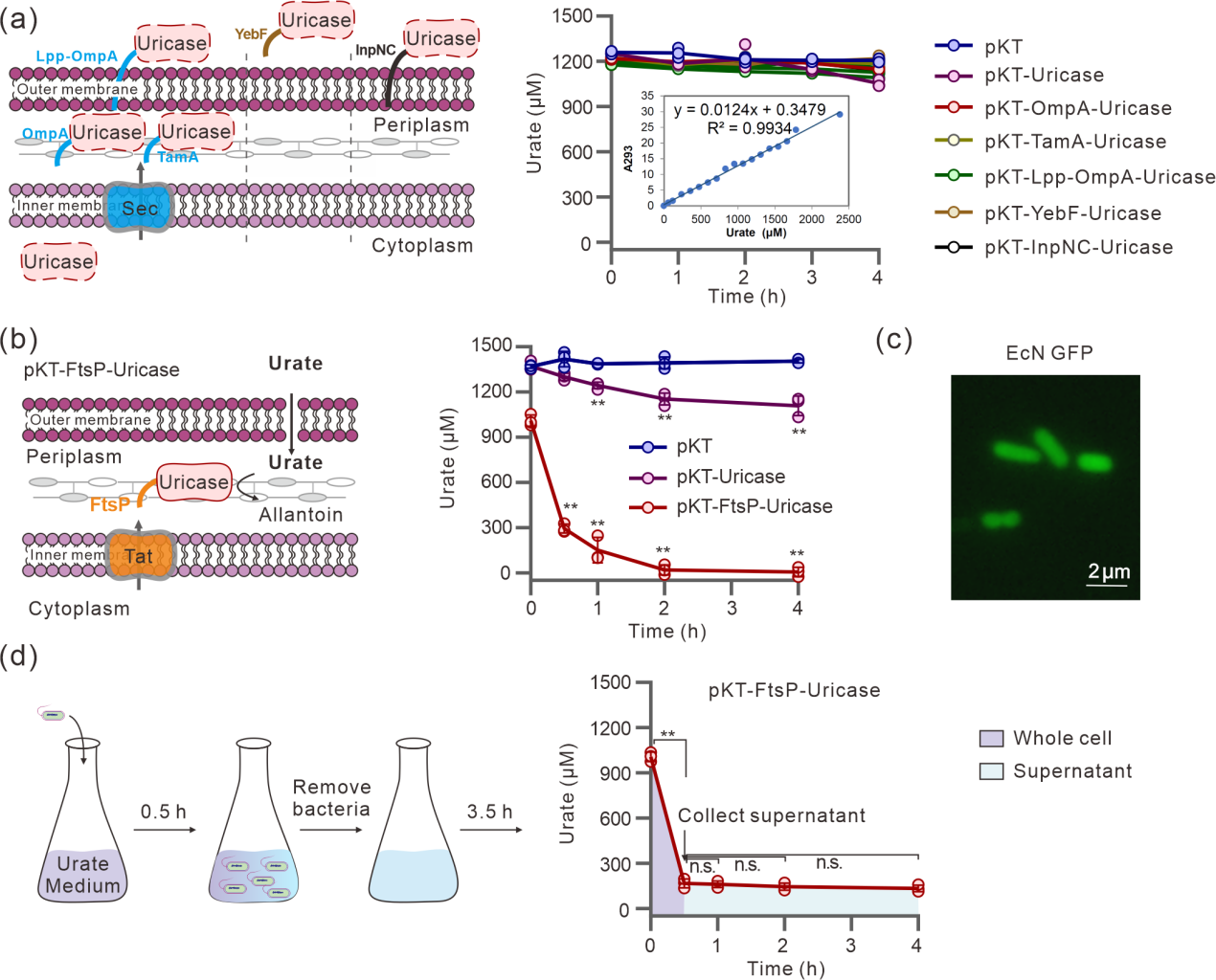


**Figure S1** Uricase activities of the recombinant strains expressing uricase gene fused with different signal peptides. (a) Overview of the proposed locations of uricase fused with different signal peptides (left) and activities of the corresponding strains in degrading urate *in vitro* (right). The standard curve between the A_293_ values and urate concentrations is shown as an insert, eighteen points of different concentrations of urate, performed in triplicate samples. (b) Diagram of periplasmic expression of uricase driven by the FtsP peptide through the Tat secretion system (left), and the urate degradation activities of the strains *in vitro* (right). (c) Localization of GFP without the FtsP signal peptide. (d) Uricase activities of whole cell or supernatant from the EcN strain expressing FtsP-uricase fused protein. Data were from triplicate independent bacterial cultures; bars indicate the mean ± SD based on two-tailed unpaired Student’s t test. (**P < 0.01).


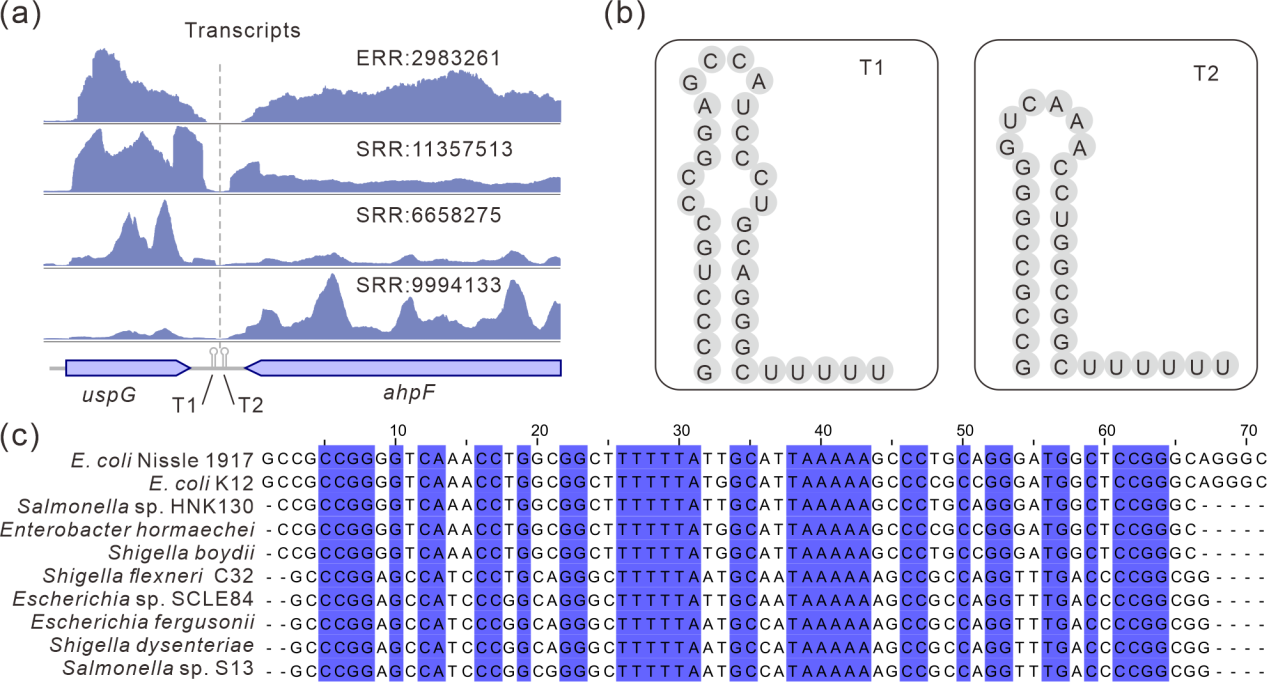


**Figure S2** Insulated site located between the *uspG* and *ahpF* genes in *E. coli.* (a) Normalized RNA levels in regions covering the *uspG* and *ahpF* genes from different studies. The site for insertion of the *ftsP-uricase* fragment in the EcN genome is shown at the bottom. (b) RNA secondary structures of the T1 and T2 terminators predicted by RNAfold service (<http://beagle.bio.uniroma2.it/>). (c) Multisequence alignments of the insulated site by ClustalW ([Multiple Sequence Alignment - CLUSTALW (genome.jp)](https://www.genome.jp/tools-bin/clustalw)).


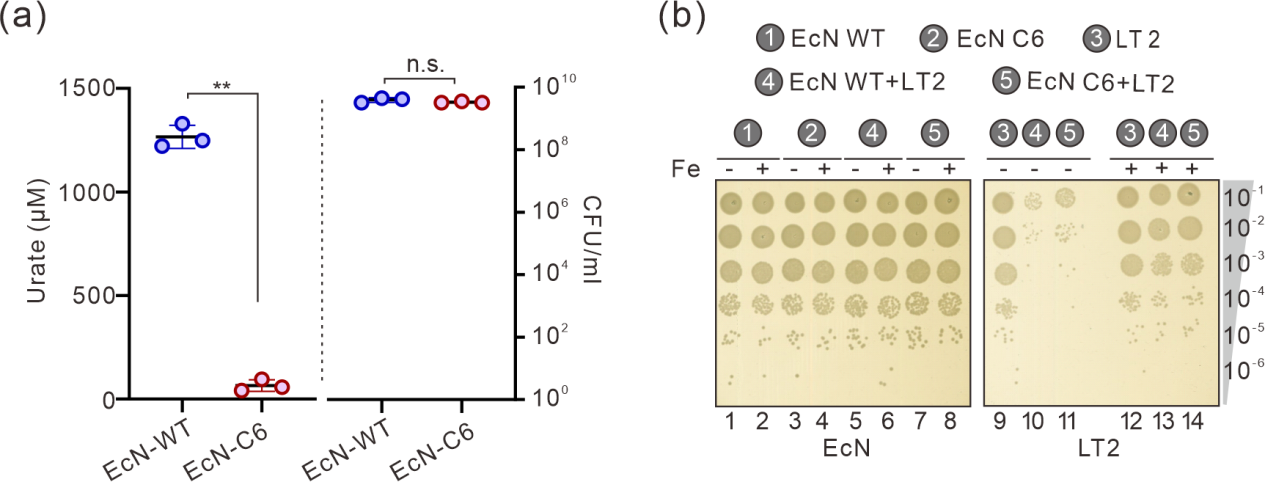


**Figure S3** Influences of uricase expression on the characteristics of EcN C6. (a) Comparisons of urate concentrations (left) and CFUs (right) of EcN WT and C6 after incubated in MU medium for one hour. Data represent measurements from three independent bacterial cultures, bars show the mean ± SD using two-tailed unpaired Student’s t-test. (**P < 0.01). (b) Competitive growth assays of EcN WT, C6 and *S*. Typhimurium LT2.


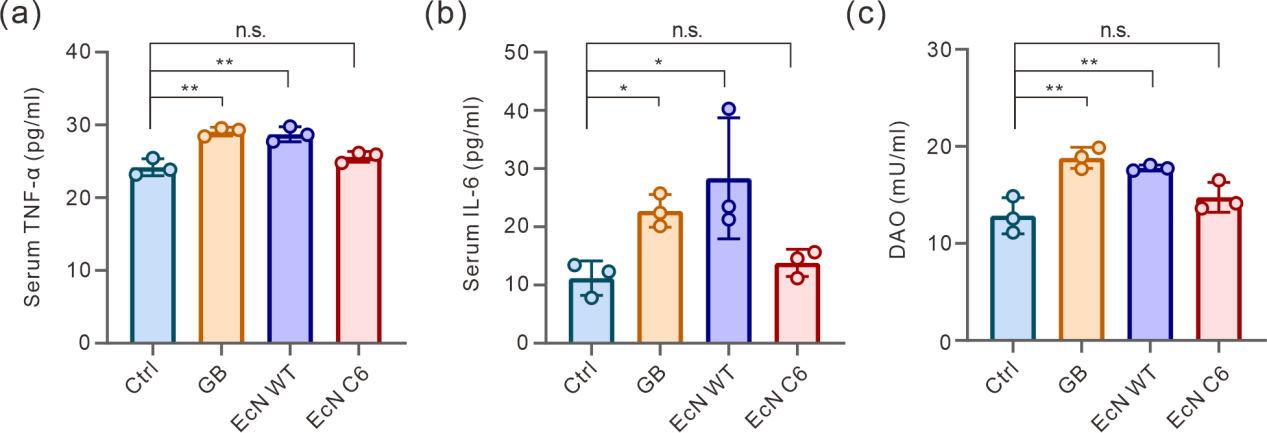


**Figure S4** Levels of serum TNF-α (a), IL-6 (b) and DAO (c) with different treatment in hyperuricemia rats. Data represent measurements from three independent serum samples, bars show the mean ± SD using two-tailed unpaired Student’s t-test, **P < 0.01; *P < 0.05.


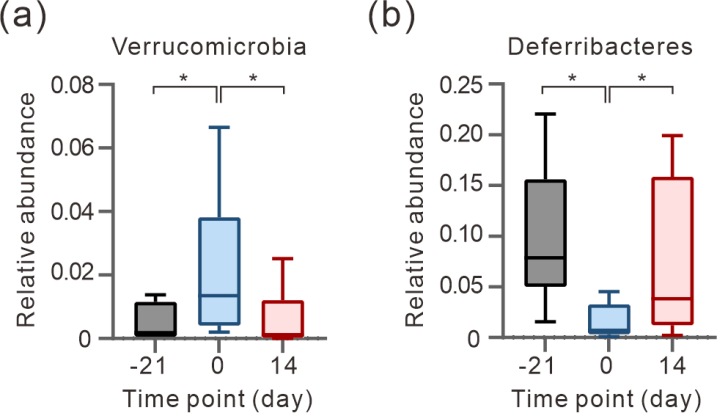


**Figure S5** Relative abundance of *Verrucomicrobia* (a) and *Deferribacteres* (b) in the gut microbiota of the EcN C6-administrated rats at -21, 0 and 14 days (n = 10). Data represent measurements from ten independent stool samples, bars show the mean ± SD using two-tailed unpaired Student’s t-test, *P < 0.05.


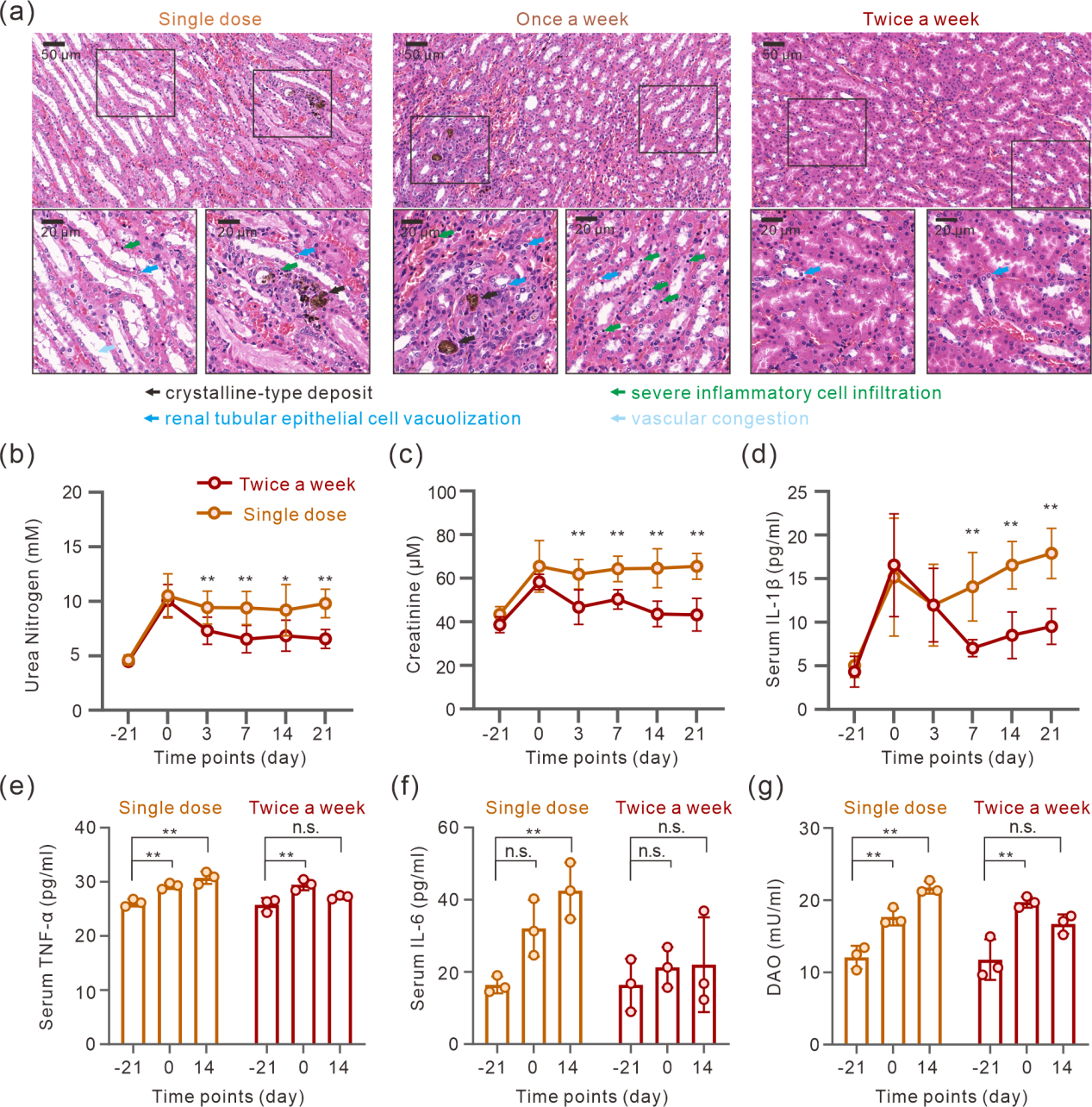


**Figure S6** Administration of EcN C6 twice per week alleviates the urate-associated inflammation. (a) Representative renal tissue sections with hematoxylin and eosin staining. Scale bars, 50 μm or 20 μm. Levels of serum urea nitrogen (b), creatinine (c), IL-1β levels (d), TNF-α (e), IL-6 (f) and DAO (g) in hyperuricemia rats with oral administration different dosage EcN C6. Data represent measurements from three independent serum samples, bars show the mean ± SD using two-tailed unpaired Student’s t-test, **P < 0.01; *P < 0.05.
